# Anti-Biofilm Performance of Resin Nanopillars Inspired from Cicada Wing Surface for *Staphylococcus* spp.

**DOI:** 10.3390/biomimetics9120739

**Published:** 2024-12-04

**Authors:** Satoka Matsumoto, Hiroaki Tatsuoka, Miki Yoshii, Toshihiro Nagao, Tomohiro Shimizu, Shoso Shingubara, Shigemitsu Tanaka, Takeshi Ito

**Affiliations:** 1 Graduate School of Science and Engineering, Kansai University, 3-3-35 Yamatecho, Suita 564-8680, Osaka, Japan; 2 Osaka Research Institute of Industrial Science and Technology, 1-6-50 Morinomiya-1, Joto-ku, Osaka-City 536-8553, Osaka, Japan

**Keywords:** anti-biofilm, nanopillar, thermal nanoimprint lithography, *Staphylococcus* spp.

## Abstract

The increase in infections derived from biofilms from *Staphylococcal* spp. prompted us to develop novel strategies to inhibit biofilm development. Nanoscale protrusion structures (nanopillars) observed on the wings of dragonflies and cicadas have recently gained notable attention owing to their physical, antimicrobial, and bactericidal properties. Thus, they are not only expected to reduce the damage caused by chemical antimicrobial agents to human health and the environment, but also to serve as a potential countermeasure against the emergence of antimicrobial-resistant bacteria (ARB). In this study, we evaluated the anti-biofilm effects of cyclo-olefin polymer (COP) nanopillars by changing the wettability of surfaces ranging in height from 100 to 500 nm against *Staphylococcus* spp., such as *Staphylococcus aureus* NBRC 100910 (MSSA), *Staphylococcus aureus* JCM 8702 methicillin-resistant *S. aureus* (MRSA), and *Staphylococcus epidermidis* ATCC 35984. The results clearly show that the fabricated nanopillar structures exhibited particularly strong biofilm inhibition against MRSA, with inhibition rates ranging from 51.2% to 62.5%. For MSSA, anti-biofilm effects were observed only at nanopillar heights of 100–300 nm, with relatively low hydrophobicity, with inhibition rates ranging from 23.9% to 40.8%. Conversely, no significant anti-biofilm effect was observed for *S. epidermidis* in any of the nanopillar structures. These findings suggest that the anti-biofilm properties of nanopillars vary among bacteria of the same species. In other words, by adjusting the height of the nanopillars, selective anti-biofilm effects against specific bacterial strains can be achieved.

## 1. Introduction

Microorganisms attach to solid surfaces and develop biofilms that protect against external stresses, and are a major cause of healthcare-associated infections [1,2,3]. Additionally, most cells in the biofilm matrix are in a dormant state or replicate slowly; therefore, the minimum bactericidal concentration (MBC) of antimicrobial agents required to eradicate them is higher than the amount required for planktonic cells [4]. Therefore, regular cleaning and disinfection procedures often fail to remove them adequately [5,6]. Medical devices, including implants, such as urinary stents, catheter tubes, pacemakers, artificial blood vessels, and artificial valves, use various biocompatible materials, such as polymers and ceramics. Medical devices designed to replace parts of the human body functionally and structurally must be biocompatible. Several researchers have attempted to enhance biocompatibility by modifying the surface properties of these materials; however, bacterial adhesion and biofilm formation on these materials continue to pose notable challenges. Biofilms that form on implants not only interfere with the function of the implants, but also account for 60–80% of the cause of healthcare-related infections, leading to substantial economic and human costs. Therefore, measures must be taken to prevent bacteria from adhering to implant surfaces and to kill bacteria when they come in contact with surfaces.

Currently, most countermeasures against biofilm function are based primarily on chemical mechanisms. Chemical methods include the use of bleach or sanitizing detergents to chemically interact with and kill bacteria. However, they are clearly inappropriate for use in biological environments, as many of them are highly toxic and can damage the material surface on which they are used. Other methods include incorporating or embedding antibiotics into the surface treatment layer [7,8], or coating the surface with polymers or antimicrobial peptides to reduce the adhesion of bacteria to the material or to disrupt bacterial cell membrane functions [9,10]. However, the long-term use of antibiotics may lead to outbreaks of drug-resistant bacteria, which make infections intractable and chronic [11,12,13,14]. In addition, with the use of antimicrobial peptides and polymer surface coatings, to maintain consistent antimicrobial activity owing to issues such as coating exfoliation and chemical instability in biological environments is challenging. In recent years, physical disinfection methods have been explored as new strategies to replace chemical methods. The most anticipated approach is to provide nano- and sub-micrometer-scale structures on material surfaces to inhibit bacterial adhesion and growth. In designing such antimicrobial surfaces, several studies have attempted to use biomimetics as a means of altering the surface properties. Biomimetics is a technology that mimics and utilizes functional structures developed through the evolution of organisms to adapt to various environments. Over the past few years, several new technologies and materials based on biomimetics have been investigated, focusing on properties such as antifouling, self-cleaning, antimicrobial, radiation, and moisture resistance inspired by organisms, such as lotus leaves, dragonflies, and geckos [15,16,17,18]. Sharks and cicadas are representative examples of target species to be mimicked. Shark scales have a rational structure for infection prevention [19]. Plates made from silicone elastomers that mimic sharkskin scales have antimicrobial effects against various pathogens [20,21]. Furthermore, the technology has been commercialized under the brand name “Sharklet” and is now used in medical devices as well as smaller items, such as mouse pads and cell phone covers. Nanopillar structures on the wings of cicadas have been reported to exhibit bactericidal activity by physically disrupting bacterial cell membranes upon contact [22]. The bactericidal mechanisms of nanopillars remain unclear and several researchers have attempted to elucidate these mechanisms using various materials and structures. Ivanova et al. [23] showed that when bacterial cells attached to nanopillar structures, the cell membrane stretched between the pillars. Cell death was observed when the degree of stretching was sufficient to damage the cell membrane. Bandara et al. [24] proposed that bacterial membrane damage was initiated by a combination of strong adhesion between nanopillars and the bacterial EPS layer, as well as shear force when immobilized bacteria attempted to move on the nanostructure. The cell membrane growth of bacteria attached to the nanostructures has been suggested to be inhibited, which causes autolysis and the generation of reactive oxygen species, which might show a bactericidal effect [25,26,27]. This bactericidal effect was due to the mechanical interaction between the nanopillar structures and bacteria, which is expected to create an alternative bactericidal surface to chemical methods.

In previous studies, we reported the successful fabrication of nanopillar structures on cyclo-olefin polymer (COP) surfaces using thermal nanoimprint lithography. Notably, these nanopillar structures showed high antimicrobial activity against *Staphylococcus aureus* (*S. aureus*), a Gram-positive bacterium [28]. In general, Gram-positive bacteria are less sensitive to nanopillar structures than Gram-negative bacteria [23,29,30] because the mechanical energy required to rupture the cell membrane is generally higher in Gram-positive bacteria owing to their cell rigidity, which is determined by the elasticity, thickness, and chemical composition of the cell wall. However, the nanopillar structures used in a previous study showed better antimicrobial activity against *S. aureus* compared with *Escherichia coli* (*E. coli*). Furthermore, we confirmed that the nanopillar structures on the COP film surface showed anti-biofilm properties against *E. coli*, *Pseudomonas aeruginosa* (*P. aeruginosa*), and *S. aureus* [31].

*Staphylococcus* spp., such as *S. aureus* and *Staphylococcus epidermidis*, are opportunistic pathogens commonly found on human skin, which become pathogenic under certain conditions. They are the primary cause of orthopedic implant infections [32,33]. Additionally, methicillin-resistant *S. aureus* (MRSA) is one of the most clinically significant drug-resistant bacteria in *Staphylococcus* spp. because MRSA causes intractable infections and outbreaks in hospitals [14,34]. As our COP nanopillars showed antimicrobial and anti-biofilm activities against *S. aureus*, they have the potential to solve these problems. In this study, we fabricated COP nanopillars of varying heights to evaluate their effect on biofilm formation by three strains of Gram-positive opportunistic pathogens: *S. aureus* NBRC 100910 (MSSA), *S. aureus* JCM 8702 (MRSA), and *S. epidermidis* ATCC 35984.

## 2. Materials and Methods

### 2.1. Materials

Al plates (purity: 99.99%, 25 mm × 25 mm, 500 µmt) were obtained from Nikkal Shoko Co., Ltd. (Tokyo, Japan). Acetone, ethanol, perchloric acid, oxalic acid, phosphoric acid, and chromium oxide (VI) were obtained from Fujifilm Wako Pure Chemical Corporation (Osaka, Japan). Crystal violet (CV) solution was purchased from Kishida Chemical Co., Ltd. (Osaka, Japan). A COP film (ZF16, 100 µmt) was obtained from ZEON Corporation (Tokyo, Japan). Tryptic soy broth (TSB) medium was purchased from Becton Biosciences (Franklin Lakes, NJ, USA).

### 2.2. Anodized Aluminum Oxide (AAO) Template Fabrication Methods

**Figure 1:**
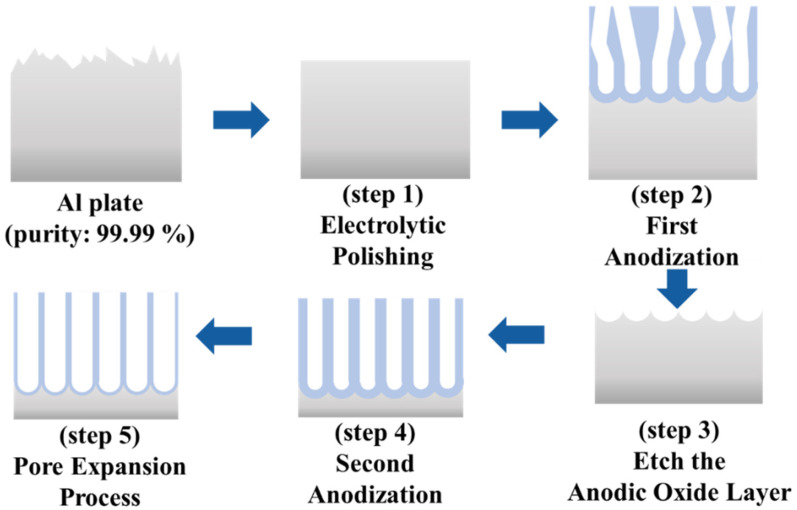
Schematic of preparing AAO nanohole array for nanoimprint template.

The COP nanopillars were fabricated by transferring periodic nanohole arrays, which were fabricated to anodize Al plates called AAO templates, onto the COP films using thermal nanoimprint lithography. AAO is an oxide thin film formed on the surface of aluminum (Al) by applying a constant voltage to an acidic electrolyte solution, where Pt and Care used as the anode and cathode, respectively. Under anodic oxidation conditions, nanoholes can be easily produced because they are self-organized. Due to this feature, AAOs have recently attracted attention for applications in high-density magnetic recording media, various optical devices, sensors, and batteries [35]. The AAO template used in this study was fabricated using a two-step anodization and pore-widening process [36]. Figure 1 shows the fabrication procedure for the AAO template.

Initially, the aluminum substrate was electropolished in a preheated solution of ethanol and perchloric acid to remove surface roughness and flatten the substrate (Step 1). Next, anodization was performed in an oxalic acid solution to create regularly arranged indentations on the Al surface (Step 2). After the first anodization step, the anodized layer was etched by immersion in a mixed solution of phosphoric acid and chromium oxide (VI) (Step 3). The second anodization step was performed using the indentations created during the first anodization as the starting point to form periodic nanoholes (Step 4). Finally, pore expansion was performed by immersing the specimens in a phosphoric acid solution (Step 5). The pitch and depth of the nanoholes were adjusted during the first and second anodization steps, respectively, and the diameter of the nanoholes was controlled through the pore expansion process. In other words, the dimensions of the nanoholes can be controlled by varying the fabrication conditions [37,38]. The conditions used to fabricate the AAO templates are listed in Table 1.

### 2.3. Transferring the Nanoholes on the AAO Template to COP Film

The fabricated AAO template was transferred onto a COP film using thermal nanoimprint lithography. Thermal nanoimprint lithography is a technique in which a template pattern is transferred onto a film by applying pressure and heat at a temperature at which the film softens. This method has the advantages of low cost and low energy consumption. The fabricated AAO template was transferred onto a 50 mm × 50 mm COP film. First, the AAO template and COP film were preheated above their glass transition temperature (Tg) = 163 °C because the film cannot plastically deform below its Tg. Subsequently, heating and pressurization were performed simultaneously (180 °C, 4 MPa, 10 min). Cooling was then performed while maintaining the pressure. Finally, the pressure was released and the AAO template and COP film were removed. An electric hot press (G-12RS; Orihara Industrial Co., Ltd., Tokyo, Japan) was used for pressing and temperature control. To facilitate release of the imprinted film, a release agent (Mold Spat 6847, AGC Seimi Chemical Co., Ltd., Kanagawa, Japan) was applied to the fabricated AAO template prior to thermal nanoimprinting.

### 2.4. Scanning Electron Microscopy Observations

The topology of the specimen surfaces was observed using scanning electron microscopy (SEM; SM-7500F, JEOL Ltd., Tokyo, Japan) at an acceleration voltage of 2 kV. A thin Pt layer was sputtered on the specimen surface to increase the conductivity of the film.

### 2.5. Plasma Treatment

Plasma treatment at a flow rate of 50 L/min (gas: air), power output of 620 W, and treatment time of 100 s was applied to each specimen using a soft plasma etching system (SEDE-GE, Meiwafosis Co., Ltd., Tokyo, Japan). In this way, hydrophilic specimens were prepared to evaluate the dependence of anti-biofilm activity on surface wettability because the COP film is hydrophobic.

### 2.6. Contact Angle Measurements

The static contact angle for aqueous solutions was measured under ambient conditions of 23 °C using a contact angle meter (DMO-502, Kyowa Surfaces Science, Saitama, Japan). We measured the static contact angles of water, TSB medium, and bacterial supernatants on nanostructured surfaces. In the case of hydrophobic specimens, 8 µL of each solution was dropped and the contact angle was measured using the Young–Laplace method. This method calculates the surface tension by fitting the Young–Laplace equation to the droplet contour shape and density difference values through image processing. For hydrophilic specimens, 2 µL of each liquid was dropped, and the contact angle was measured using the θ/2 method. The θ/2 method calculates the contact angle by assuming that the droplet contour is part of a circle, finding the angle to the solid surface of a straight line connecting the left and right end points of the droplet and the apex, and doubling this angle.

### 2.7. Cultivation of Microorganisms

*S. aureus* NBRC 100910 (MSSA), *S. aureus* JCM 8702 (MRSA), and *S. epidermidis* (ATCC 35984) were used for anti-biofilm tests. Each strain was pre-cultured in tryptic soy broth (TSB) medium (casein-pancreatic digested peptone: 17 g/L, soybean-papain digested peptone: 3 g/L, sodium chloride: 5 g/L, dipotassium phosphate: 2.5 g/L, glucose: 2.5 g/L) overnight at 37 °C with shaking at a speed of 250 rpm. The bacterial suspension for the anti-biofilm test was adjusted to 10^3^–10^4^ CFU/mL from the preculture broth using 100% TSB medium. For the preparation of bacterial supernatant used in the contact angle measurements, the preculture was adjusted to 10^7^–10^8^ CFU/mL in TSB medium. The adjusted bacterial suspension was then incubated under shaking conditions at 37 °C and 250 rpm for 6 h. The bacterial supernatants were recovered according to the methodology described by Sun et al. [39]. After cultivation, the bacterial culture was subjected to ultrasonic treatment at 28 kHz for 2 min, followed by centrifugation at 8000× *g* for 10 min. The supernatants were decanted into sterile tubes. After filtering bacterium cells via a 0.45 μm filter, the supernatant was stored at −30 °C until the test.

### 2.8. Anti-Biofilm Assay in COP Nanopillars

The specimens were prepared using three hydrophobic and hydrophilic pillars of different heights (100, 300, and 500 nm). The flat COP film was hydrophobic and was used as the control (FLA). The specimen setup is listed in Table 2. Hydrophobic specimens with heights of 100, 300, and 500 nm were named H100, H300, and H500, respectively. Hydrophilic specimens with heights of 100, 300, and 500 nm were prepared by the plasma treatment of the hydrophobic specimens and named pl_H100, pl_H300, and pl_H500, respectively.

For the anti-biofilm assay, each specimen was cut into 10 mm × 10 mm squares. Each specimen was fixed to the bottom of a 24-well plate by utilizing a 1.5% agar solution. A quantity of 1.25 mL of the bacterial suspension was dispensed into each well (in which the specimens were placed) and incubated at 37 °C for 48 h for biofilm formation. After incubation, the specimens were rinsed with deionized water to remove excess stain and planktonic bacteria. They were then dried, and the amount of biofilm was assessed using crystal violet (CV) staining. This method quantified the amount of biofilm based on CV dye adsorption because of the linear correlation between CV dye adsorption and the amount of biofilm formed [40]. In this study, 1 mL of 0.1% CV solution was added to a well containing the specimen and immersed for 30 min to stain the biofilm. Excess CV solution was removed using deionized water. To quantify biofilm production, CV was re-dissolved in solution (95% ethanol, 1% acetic acid) for 30 min, and absorbance at 595 nm was measured using a microplate reader (Nivo 3F, Perkin Elmer, Shelton, CT, USA). Welch’s *t*-test was performed for statistical analysis of the obtained data. The results were considered statistically significant when the *p*-value was less than 0.05. For results that were significantly different in the Welch’s *t*-test, the percentage of biofilm inhibition was calculated using the following equation:

(1) $$\%\;Inhibition=\left[\frac{{OD}_{pillar}-{OD}_{FLA}}{{OD}_{FLA}}\right]\times 100$$

Biofilm inhibition was rated between 0 and 100%. Values below 0% were categorized as biofilm growth enhancement, between 0 and 50% indicated weak anti-biofilm activity, and above 50% represented strong biofilm inhibition.

## 3. Results

### 3.1. Fabricated COP Nanopillars

**Figure 2:**
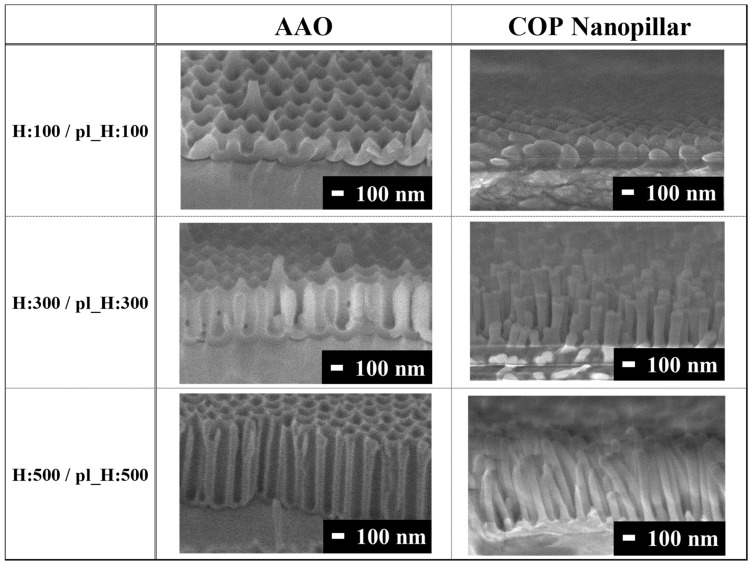
SEM images of the fabricated AAO templates and COP nanopillars.

**Figure 3:**
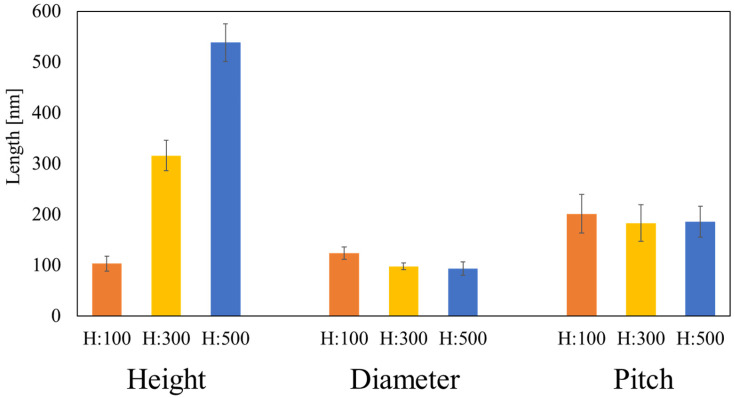
Dimensions of the fabricated nanopillars. Orange, yellow, and blue represent the specimens with H:100, H:300, and H:500, respectively.

COP nanopillar films of different heights were prepared using thermal nanoimprint lithography. The AAO templates were fabricated such that the nanoimprinted nanopillar structures (H:100, H:300, and H:500) had the same diameter, pitch, and height. The SEM observations confirmed that the nanoholes in these templates were highly vertical (Figure 2). The SEM images of the structures imprinted on the COP film using these templates are also shown in Figure 2. The average heights, diameters, and pitch sizes of the nanopillars are presented in Figure 3 (n = 5). Cross-sectional SEM images (see Figure S1 in Supporting Information) were analyzed to measure their dimensions using ImageJ (version 1.50 i) software. Additionally, the statistical analysis was performed using Microsoft Excel. The H:100 nanopillars had a diameter of 124.0 ± 12.1 nm, pitch of 201.6 ± 38.5 nm, and height of 103.2 ± 14.3 nm; the H:300 nanopillars had a diameter of 98.1 ± 6.48 nm, pitch of 183.1 ± 36.3 nm, and height of 316.0 ± 29.8 nm; and the H:500 nanopillars had a diameter of 93.5 ± 13.6 nm, pitch of 185.8 ± 30.3 nm, and height of 538.5 ± 37.5 nm. The nanopillars were well transferred and were found to stand on their own in a relatively orderly fashion.

### 3.2. Characterization of Wettability of Specimens

**Figure 4:**
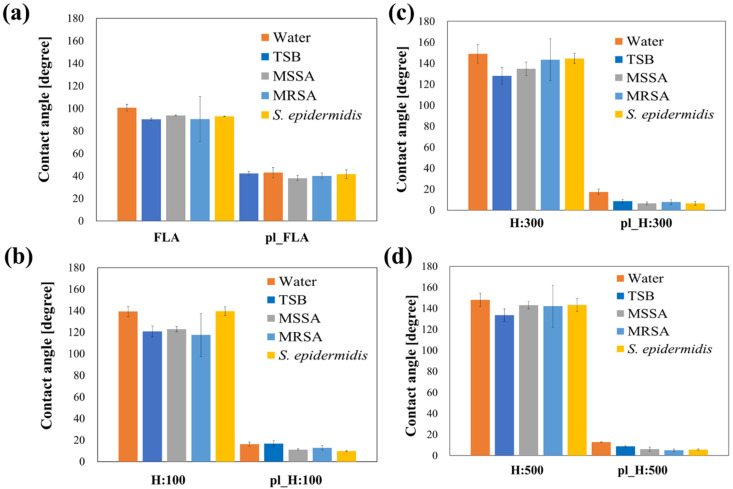
Contact angles of test specimen surfaces with/without plasma treatment for five solutions. The specimen height ranged from 100 to 500 nm. (**a**) FLA, (**b**) H:100, (**c**) H:300, (**d**) H:500.

The static contact angles of the COP films with nanopillars of three different heights (H:100, H:300, and H:500) and flat COP films (FLA) were measured before and after plasma treatment to evaluate the effect of surface wettability on biofilm formation. Plasma treatment was performed to transform hydrophobic COP films into hydrophilic surfaces. The results are shown in Figure 4.

When water was used as the test liquid, the hydrophobic specimens with nanopillar structures exhibited significantly higher contact angles (139.2°–149.0°) compared to the flat surface (100.6°). The water contact angle (WCA) increased with nanopillar height up to 300 nm but remained unchanged for heights exceeding 300 nm. In contrast, plasma-treated specimens with nanopillar structures showed significantly reduced contact angles (12.7°–17.4°) compared to the flat surface (42.1°). Figure S2 shows water contact angle images on COP nanopillars and flat COP films.

Similar trends were observed when TSB medium or bacterial supernatants were used as test liquids. Hydrophobic specimens with nanopillar structures exhibited higher contact angles than the flat surface. For plasma-treated specimens, the contact angles on all nanopillar structures were lower than those on the flat surface. Moreover, in most specimens, the contact angles with TSB medium and bacterial supernatants tended to be lower than those with water.

### 3.3. Anti-Biofilm Performance of the Hydrophobic COP Nanopillars

**Figure 5:**
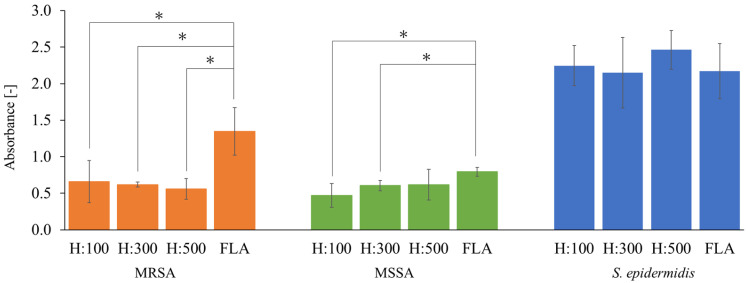
Absorbance corresponding to the amount of biofilm formation after biofilm formation tests on flat COP films and COP films with nanopillar structures. All specimens are hydrophobic. Each datum represents the mean (n = 12) and error bars represent standard deviations. The statistical differences were assessed using Welch’s *t*-test where the *p*-value indicates the level of statistical difference, and a *p* value less than 0.05 was considered statistically significant. * indicates significant difference.

Figure 5 shows the results of biofilm formation on hydrophobic COP nanopillars. The anti-biofilm performance of the hydrophobic COP nanopillars was evaluated using CV staining. Example images after the CV staining against MRSA are shown in Figure S3. In the case of MRSA, the results showed that the absorbance value of the flat surface (FLA) was 1.3 ± 0.33, whereas for the nanopillar surface it was 0.66 ± 0.29 for H:100 (*p* = 0.019 < 0.05), 0.62 ± 0.035 at H:300 (*p* = 0.021 < 0.05), and 0.56 ± 0.14 (*p* = 0.011 < 0.05) for H:500, confirming a significant anti-biofilm effect. The biofilm inhibition rates were 51.2% for H:100, 54.0% for H:300, and 58.4% for H:500.

In the case of MSSA, significant biofilm suppression was observed on H:100 (Abs. = 0.47 ± 0.16, *p* = 0.020 < 0.05) and H:300 (Abs. = 0.61 ± 0.072, *p* = 0.007 < 0.05), compared to FLA (Abs. = 0.80 ± 0.060). The inhibition rates were 40.8% for H:100 and 23.9% for H:300. However, for H:500, no significant suppression was observed (Abs. = 0.62 ± 0.21, *p* = 0.21 > 0.05).

For *S. epidermidis*, no significant anti-biofilm effect was observed for any nanopillar structure, with absorbance values comparable to those of the FLA surface.

### 3.4. Anti-Biofilm Effect Performance of the Hydrophilic COP Nanopillars

**Figure 6:**
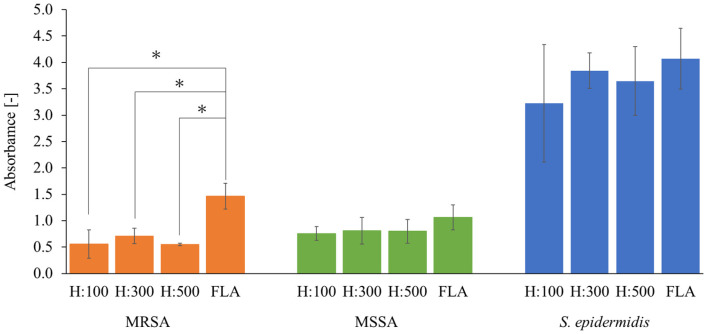
Absorbance corresponding to the amount of biofilm formation after the biofilm formation test on a flat COP film and a COP film with a nanopillar structure. All specimens were hydrophilized by plasma treatment. Each datum represents the mean value (n = 12) and error bars represent standard deviation. The statistical differences were assessed using Welch’s *t*-test where the *p*-value indicates the level of statistical difference, and a *p* value less than 0.05 was considered statistically significant. * indicates significant difference.

Figure 6 shows the results of biofilm formation on hydrophilic COP nanopillars. Hydrophilic COP nanopillars showed significant anti-biofilm properties against MRSA; these properties were the same as the those of the hydrophobic nanopillars. The absorbance values at 48 h were 0.56 ± 0.27 (*p* = 0.0024) for H:100, 0.71 ± 0.14 (*p* = 0.0031) for H:300, and 0.55 ± 0.023 (*p* = 0.0050) for H:500, significantly lower than the 1.5 ± 0.24 of FLA. The biofilm inhibition rates were 62.0% for H:100, 51.6% for H:300, and 62.5% for H:500. In contrast, for MSSA, the nanopillar samples showed a slight reduction in biofilm formation compared to FLA (1.1 ± 0.24), but these reductions were not statistically significant at any nanopillar height. Similarly, no significant biofilm inhibition was observed in *S. epidermidis* in the nanopillar samples compared to FLA (4.1 ± 0.58).

## 4. Discussion

This study evaluated the relationship between the height of nanopillar structures formed on COP films and biofilm formation by three strains of *Staphylococcus* bacteria under varying hydrophilic and hydrophobic surface conditions. The height of the nanopillars is considered as one of the key factors influencing the antimicrobial effects on bacteria. However, several aspects of the antimicrobial mechanisms remain unclear. Currently, the physical deformation and rupture of bacterial cells caused by contact with nanopillars are regarded as the primary factors contributing to bactericidal mechanisms [24,41,42]. Thus, the number and area of contact points with bacterial cells are important. The pitch size and diameter of nanopillars are critical factors that influence bacterial cell membrane deformation, contact area, and pressure distribution. Previous studies have reported that reducing the pitch size of nanostructures enhances antimicrobial effects [43,44,45]. Watson et al. [46] predicted through theoretical modeling that the pitch must not exceed the diameter of the bacterial cell for the nanopillars to exert sufficient stress on the bacterial membrane for antimicrobial effects, including bactericidal effects. Additionally, Xiang et al. [47] showed that bacteria tend to become trapped between pillars without affecting bacterial growth when the pillar spacing is wider than the bacterial size. As the typical diameter of *S. aureus*, a model Gram-positive bacterium, is approximately 1 µm, it is unlikely that nanopillar structures with a pitch size greater than 1 µm would exhibit bactericidal properties. Furthermore, Pegodin et al., using computer simulation, reported that the diameter and height of nanopillars influence the contact area with bacteria and may cause membrane damage [48]. To primarily investigate height-dependent anti-biofilm effects, this study fixed the pitch size at 200 nm and the diameter at 100 nm, and the nanopillars with three different heights (100, 300, and 500 nm) were fabricated.

The results revealed a significant difference in nanopillar height-dependent biofilm formation in MSSA cultured on hydrophobic surfaces. In particular, the amount of biofilm was significantly lower in the H:100 (*p* = 0.02) and H:300 (*p* = 0.007) specimens than on the flat plate (FLA). The biofilm inhibition rates at H:100 and H:300 were 40.8% and 23.9%, respectively. This means that the smaller nanopillars tended to show higher anti-biofilm activity. This anti-biofilm effect could be associated with the local elastic modulus (LEM) of the nanopillar structure. According to the study by Zhao et al. [49], shorter nanopillars exhibit a higher local elastic modulus (LEM), which leads to greater bactericidal property. Shorter nanopillars deform less than taller ones when subjected to horizontal forces and can store and release greater energy. This mechanism facilitates physical damage to bacterial cells, thereby enhancing their bactericidal efficacy [50]. Such physical damage can be one of the primary contributors to the bactericidal effect of the nanopillar structure.

Our previous studies confirmed that pillars with a lower height in the range of 100–300 nm tend to show higher antimicrobial activity against *E. coli* [37]. In addition, several studies have reported that polymeric nanopillar structures exhibit antibacterial activity, except those using COP. The surfaces covered with polycarbonate nanopillars having a height of 150 nm were shown to kill 3% of *E. coli*, whereas surfaces with nanopillars a height > 150 nm were able to kill approximately 90% of attached bacteria [51]. Linklater et al. [52] observed significant damage to the envelopes of *S. aureus* and *P. aeruginosa* on polymer nanostructures with heights of 120 nm and 220 nm. Additionally, although the materials were different, Ivanova et al. [53] demonstrated that the highest bactericidal activity against *P. aeruginosa* and *S. aureus* was observed at a Si nanopillar height of 360 nm among three height conditions (200 nm, 360 nm, and 420 nm). Ganjian et al. [54] reported that the 3D dimensions of metal nanopillars exhibiting bactericidal activity against *S. aureus* ranged from 70 to 100 nm in diameter, 60–200 nm in pitch, and 100–900 nm in height. The nanopillar structures fabricated in this study are within this dimensional range and exhibit antimicrobial activity. However, their antimicrobial properties remain to be fully elucidated, necessitating further investigation to confirm their potential.

On the other hand, the nanopillar structures formed on COP film in this study also contribute to the modification of surface wettability. Yuan et al. [55] demonstrated that nanostructures imparted on a material surface increase the wettability of the underlying material. In other words, the presence of nanopillar structures renders hydrophobic surfaces more hydrophobic and hydrophilic surfaces more hydrophilic. In this study, all nanopillar specimens without plasma treatment showed higher water contact angles than the flat surfaces. When these surfaces were subjected to plasma treatment to render them hydrophilic, the water contact angles of the surfaces with these structures were smaller than those of flat surfaces. Therefore, changes in wettability may also be involved in the effects of the nanopillar structures on biofilm formation. The contact angle was measured using TSB in this study. The results showed that the CA on TSB decreased by 10–20° compared to the CA for water. For the hydrophilic specimens, the contact angles decreased significantly for pl_H = 300 and pl_H = 500. This decrease in CA might be attributed to components such as tryptone in the TSB medium acting as a surfactant. Additionally, the contact angle of each specimen was measured for the 6 h bacterial supernatant of each strain. Although no significant difference in contact angle was observed between the strains in the H:300 and H:500 structures, a slight hydrophobic or hydrophilic shift in contact angle was observed in the H:100 and plaH:100 samples of the culture supernatant of *S. epidermidis* compared to the other strains. This may reflect the different composition of components in the culture supernatant of *S. epidermidis* compared to *S. aureus*, such as MSSA and MRSA, and the production or degradation of substances that alter structural wettability.

In general, moderately wettable materials with a water contact angle of approximately 90° allow bacteria to adhere more than extremely hydrophobic or hydrophilic surfaces [56,57]. Lee et al. [58] reported that cell adhesion and growth were enhanced on surfaces with a water contact angle (WCA) of 40–70°, and Dou et al. [59] showed that bacterial peptidoglycan adsorption increased on surfaces with a CA of 54–130°. Yuan et al. [55] also reported that *E. coli* adherence was the highest on surfaces with a WCA of 95° and decreased under extremely high (WCA > 115°) or low (WCA < 28°) conditions. Hydrophilic surfaces have been shown to promote the adhesion of *S. aureus* and *E. coli*, and hydrophobic surfaces attract *Pseudoxanthomonas taiwanensis* and *S. epidermidis* while reducing the adhesion of *S. aureus* and *Streptococcus mutans* [56]. These results suggest that modifying material surfaces to be super-hydrophobic or super-hydrophilic may be effective for biofilm suppression, although the WCA of the surfaces most likely to adhere differed for each bacterial species.

Notably, in this study, biofilm formation by MRSA was significantly reduced at all nanopillar heights, but no height-dependent trend was observed. In contrast, for MSSA, anti-biofilm effects were observed only in the H:100 and H:300 samples. These findings suggest that lower height nanopillar inhibited biofilm formation for both MRSA and MSSA on hydrophobic specimens, and that higher nanopillars selectively inhibited MRSA.

In contrast, no significant reduction was observed for *S. epidermidis* on any structure compared with flat surfaces. In addition, the amount of biofilm formed by *S. epidermidis* was much higher than that formed by MSSA and MRSA at the absorbance level. This could possibly be due to the fact that the *S. epidermidis* (strain 35984) used in this study is known as a high EPS-producing strain [58,59]. Hizal et al. [60] reported that EPS-producing strains of *Staphylococcus* tend to die in high numbers upon contact with nanopillars and that local cell wall stress induces EPS production. Although this tendency in *Staphylococcus* is favorable for the antimicrobial properties of the nanopillar structure, the induction of EPS production may reduce its antimicrobial efficacy. In *S. epidermidis*, “pressure-induced” EPS secretion can mask nanopillar structures, diminishing their surface characteristics and associated effects. This phenomenon was also observed in *P. aeruginosa*, where the nanopillar structure was masked by bacterial residues and EPS, thereby preventing bactericidal effects [61]. In support of this, previous studies have demonstrated antimicrobial activity against *S. aureus* and *E. coli*, but not against *P. aeruginosa* [28]. However, the lack of anti-biofilm effects observed against the high EPS-producing *S. epidermidis* strain highlighted a significant challenge in achieving broad-spectrum antibacterial efficacy. Furthermore, the variability in responses among different bacterial species suggests the necessity of optimizing surface design on an individual basis. To address this issue in greater depth, further comparative studies involving various EPS-producing strains are essential. Additionally, improving the design of COP nanopillar structures and developing novel surface modification technologies and surfaces with releasing actions [62] to prevent masking by EPS are imperative. Future studies should explore strategies to decrease the effects of EPS production, such as the introduction of antifouling coatings and the application of biochemical modifications to degrade EPS. These advancements are crucial for expanding the applicability of surface modification technologies using nanopillars for a broader range of pathogenic bacteria.

In any case, the nanopillar structure on the COP film is an effective method of surface modification with an anti-biofilm effect; however, the function of inhibiting biofilm growth in addition to inhibiting initial adhesion must be explored.

## 5. Conclusions

In this study, the anti-biofilm effects of COP nanopillars of three different heights were evaluated against *S. epidermidis*, MSSA, and MRSA. The results showed that the fabricated nanopillar structures exhibited anti-biofilm effects against *S. aureus*, with a particularly strong inhibitory effect against MRSA. Additionally, the contact angles of the COP film with nanopillars shifted toward a more hydrophobic state compared to flat surfaces, and further shifted towards hydrophilicity with plasma treatment. This suggests that the nanopillar structures used in this study hold promise as a surface modification technique to inhibit the adhesion of MRSA to the surface.

Additionally, a height-dependent anti-biofilm effect was prominently observed for MSSA on hydrophobic surfaces. MRSA exhibited significant biofilm reduction across all nanopillar heights without any height-dependent effect. For *S. epidermidis*, no anti-biofilm effects were detected for all nanopillar structures. These findings indicate that the relationship between nanopillar height and anti-biofilm activity is strongly influenced by bacterial species and surface properties. This suggests that the anti-biofilm mechanism of nanopillar structures is not universal but species-specific.

Future research will need to explore other bacterial strains with diverse cell wall properties to achieve a more comprehensive understanding of the interactions between nanopillar structures and biofilm formation.

## Figures and Tables

**Table 1:** Fabrication conditions of AAO templates.

		First Anodization	Second Anodization	Pore Expansion Process
H:100	Voltage	85 V	85 V	-
	Time	900 s	30 s	1500 s
	Solution	0.3 M Oxalic acid	0.3 M Oxalic acid	Phosphoric acid
H:300	Voltage	85 V	85 V	-
	Time	900 s	90 s	1500 s
	Solution	0.3 M Oxalic acid	0.3 M Oxalic acid	Phosphoric acid
H:500	Voltage	85 V	85 V	-
	Time	900 s	150 s	1500 s
	Solution	0.3 M Oxalic acid	0.3 M Oxalic acid	Phosphoric acid

**Table 2:** Nanopillar specimens used in this study.

Test Specimens			
Hydrophobic	H:100	Hydrophilic	pl_H:100
	H:300		pl_H:300
	H:500		pl_H:500
	FLA		pl_FLA

## Data Availability

No new data were created or analyzed in this study. Data sharing is not applicable to this article.

## Supplementary Materials

The following supporting information can be downloaded at: https://www.mdpi.com/article/10.3390/biomimetics9120739/s1, Figure S1. SEM images of the COP nanopillars, indicating the diameter, pitch, and height of the nanopillars. Figure S2. Images of evaluating water contact angles (WCAs) on the COP nanopillar and on the flat COP films with (right figures)/without (left figures) plasma treatment. Figure S3. Example images after the crystal violet (CV) staining method for each sample.
