# Supplementary material for: Anti-Biofilm Performance of Resin Nanopillars Inspired from Cicada Wing Surface for Staphylococcus spp
Source: Biomimetics (Basel). 2024 Dec 4;9(12):739. doi: 10.3390/biomimetics9120739 (PMC11673656; doi:10.3390/biomimetics9120739)
Supplement: Supplementary file 1 [file biomimetics-09-00739-s001.zip › biomimetics-3311588-supplementary.pdf]

Supporting Information

Anti-biofilm performance of resin nanopillars inspired from cicada wing surface for *Staphylococcus* spp.

Satoka Matsumoto[1], Hiroaki Tatsuoka[2], Miki Yoshii[2], Toshihiro Nagao[2],Tomohiro Shimizu[1], Shoso Shingubara[1] , Shigemitsu Tanaka[2*]and Takeshi Ito[1*]

[1]Graduate School of Science and Engineering, Kansai University, 3-3-35 Yamatecho, Suita, Osaka 564-8680, Japan

[2]Osaka Research Institute of Industrial Science and Technology, 6-50 Morinomiya-1, Joto-ku, Osaka-City, Osaka 536-8553, Japan

*Correspondence: t.ito@kansai-u.ac.jp

*Correspondence: s-tanaka@orist.jp

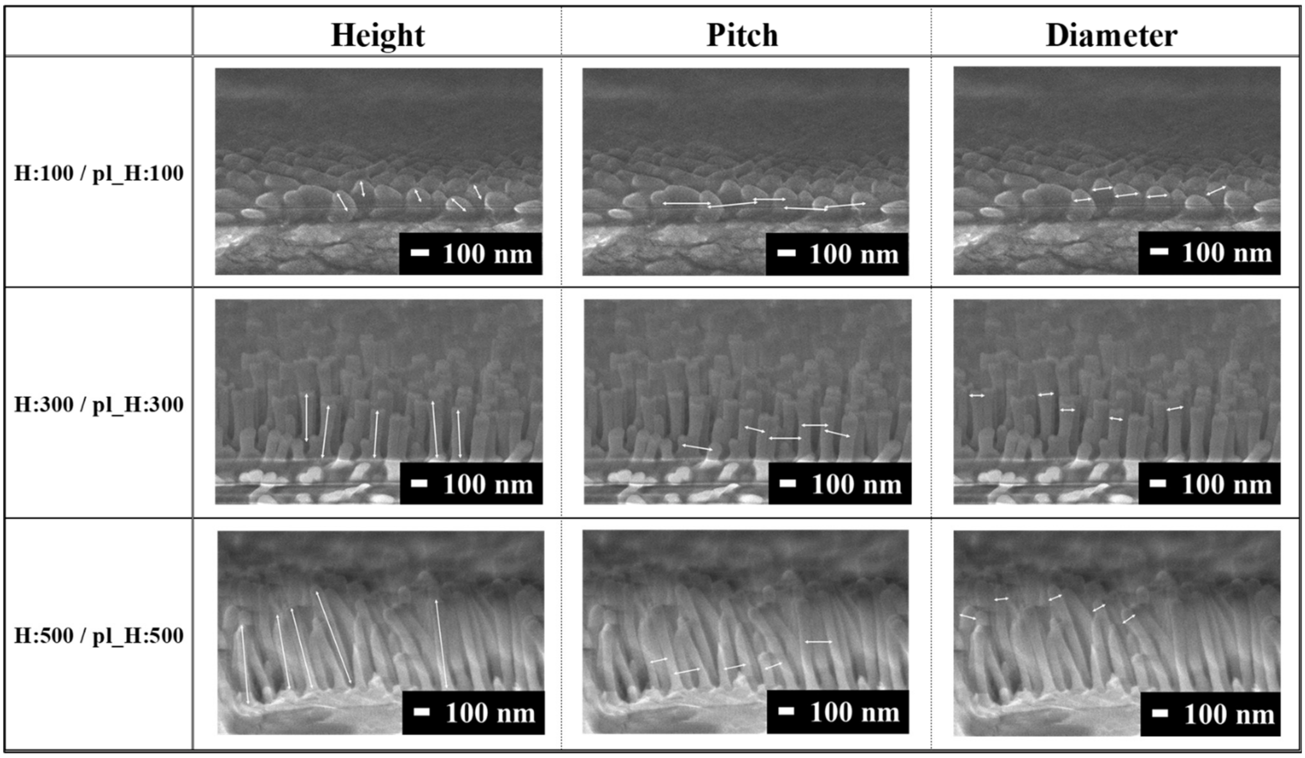

Figure S1. SEM images of the COP nanopillars, indicating the diameter, pitch, and height of the nanopillars.

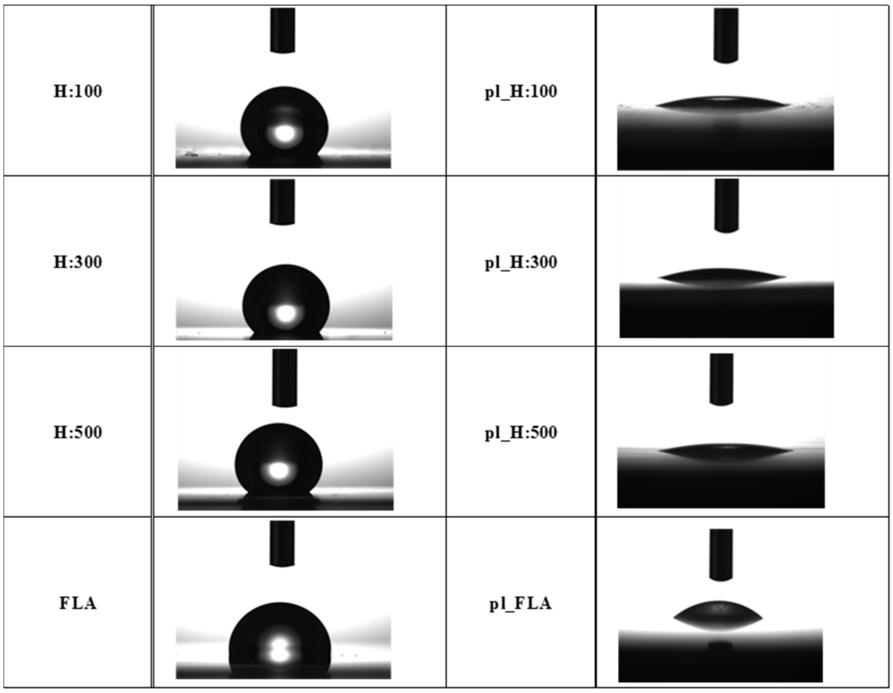

Figure S2. Images of evaluating water contact angles (WCAs) on the COP nanopillar and on the flat COP films with (right figures)/without (left figures) plasma treatment.

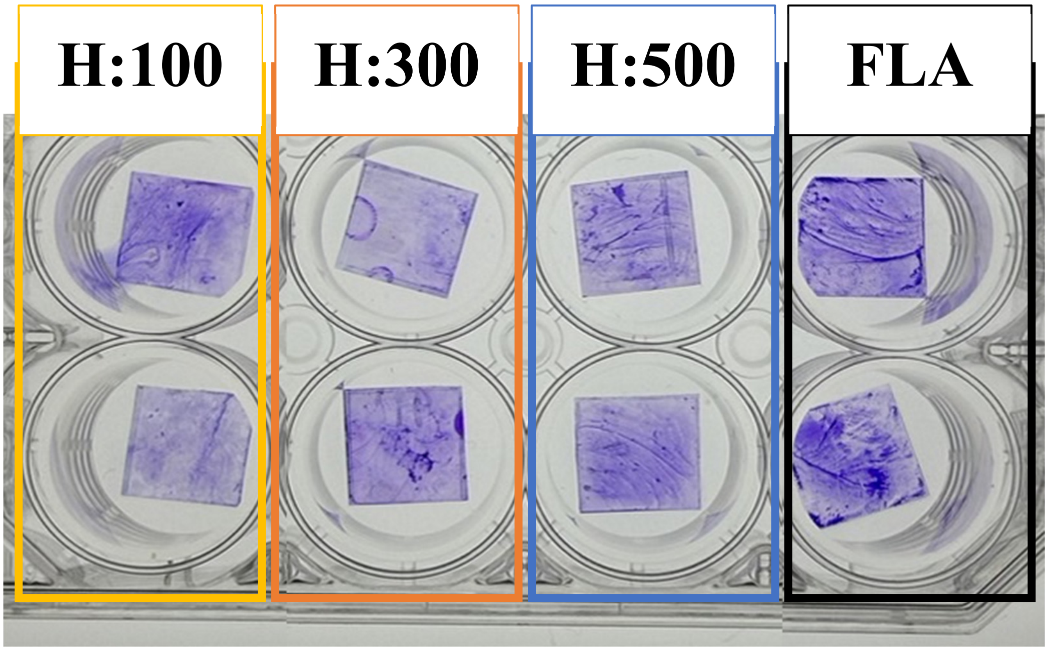

Figure S3. Example images after the crystal violet (CV) staining method for each sample.
